# Implementing a shared decision-making intervention to support treatment decisions for patients following an anterior cruciate ligament rupture — a protocol for the POP-ACLR feasibility study

**DOI:** 10.1186/s40814-024-01503-6

**Published:** 2024-05-07

**Authors:** Hayley M. Carter, David J. Beard, Charlotte Dodsley, Paul Leighton, Joshua McCallion, Fiona Moffatt, Benjamin E. Smith, Kate E. Webster, Pip Logan

**Affiliations:** 1 Physiotherapy Outpatients, Florence Nightingale Community Hospital, Level 3, Derby, DE1 2QY UK; 2 School of Medicine, Queens Medical Centre, University of Nottingham, Nottingham, NG7 2UH UK; 3 Surgical Intervention Trials Unit, NDORMS, Botnar Research Centre, University of Oxford, Windmill Road, Oxford, OX3 7LD UK; 4 Patient representative, The POP-ACLR Study, Nottingham, UK; 5 School of Health Sciences, Queen’s Medical Centre, University of Nottingham, Nottingham, NG7 2HA UK; 6 School of Allied Health, Human Services and Sport, La Trobe University, Kingsbury Drive, Bundoora, VIC 3086 Australia

**Keywords:** Anterior cruciate ligament, Shared decision-making, Intervention development, Nominal group technique, Extended normalisation process theory

## Abstract

**Background:**

Treatment for anterior cruciate ligament (ACL) rupture may follow a surgical or nonsurgical pathway. At present, there is uncertainty around treatment choice. Two shared decision-making tools have been codesigned to support patients to make a decision about treatment following an ACL rupture. The shared decision-making tools include a patient information leaflet and an option grid. We report the protocol for a mixed-methods feasibility study, with nested qualitative interviews, to understand feasibility, acceptability, indicators of effectiveness and implementation factors of these shared decision-making tools (combined to form one shared decision-making intervention).

**Methods:**

A single-centre non-randomised feasibility study will be conducted with 20 patients. Patients diagnosed with an ACL rupture following magnetic resonance imaging will be identified from an orthopaedic clinic. The shared decision-making intervention will be delivered during a clinical consultation with a physiotherapist. The primary feasibility outcomes include the following: recruitment rate, fidelity, acceptability and follow-up questionnaire completion. The secondary outcome is the satisfaction with decision scale. The nested qualitative interview will explore experience of using the shared decision-making intervention to understand acceptability, implementation factors and areas for further refinement.

**Discussion:**

This study will determine the feasibility of using a newly developed shared decision-making intervention designed to support patients to make a decision about treatment of their ACL rupture. The acceptability and indicators of effectiveness will also be explored. In the long term, the shared decision-making intervention may improve service and patient outcomes and ensure cost-effectiveness for the NHS; ensuring those most likely to benefit from surgical treatment proceed along this pathway.

**Trial registration:**

Pending registration on ISRCTN.

**Supplementary Information:**

The online version contains supplementary material available at 10.1186/s40814-024-01503-6.

## Introduction

### Background and rational

Anterior cruciate ligament (ACL) ruptures are a common musculoskeletal injury, accounting for over 20,000 knee injuries in the UK each year [1]. Once diagnosed, treatment may follow a nonsurgical, surgical, or combined pathway. To date, there have been three randomised controlled trials (RCTs) comparing surgical (ACL reconstruction [ACLR]) and nonsurgical treatment demonstrating conflicting findings and results [2–4]. At present, the evidence is uncertain, and it is not clear who is most likely to benefit from surgical or nonsurgical treatment.

A qualitative study (manuscript in preparation), exploring the experiences of patients on the surgical pathway in the NHS, revealed uncertainty with decision-making about surgery. The decision-making process was described in three ways by participants who as follows: (1) felt the decision was made for them (with limited opportunity of shared decision-making practices); (2) wanted to avoid responsibility for the decision, deferring to the opinion of experienced healthcare professionals; and (3) did not feel a balanced argument was presented to them and thus felt there was no real decision to be made (with advice favouring surgical intervention to support a return to physical activity). This reveals uncertainty amongst patients in addition to uncertainty in the evidence base on the decision-making process.

A nominal group consensus study (manuscript in preparation) produced two co-designed shared decision-making (SDM) tools to be used as an intervention package to support decision-making regarding management following an ACL rupture. Patients and key stakeholders were involved in its development ensuring the tools were based on the latest evidence and expert opinion. Stakeholders included physiotherapists (working in musculoskeletal [MSK] outpatient and orthopaedic departments) an occupational therapist (working in an MSK outpatient department specialising in vocational rehabilitation), an orthopaedic surgeon and outpatient therapy manager (who previously worked as an MSK physiotherapist and previously had an ACLR). The development process was also underpinned by the extended normalisation process theory (ENPT) to ensure factors concerning implementation of the tools were considered and embedded within the design [5]. The tools aim to ensure patients are able to make informed decisions about their treatment, and that the surgical pathway is appropriate for all those experiencing it. This novel SDM intervention is therefore ready for implementation and feasibility testing. This paper reports the protocol for the mixed-methods feasibility study.

### Objectives

**Fig. 1:**
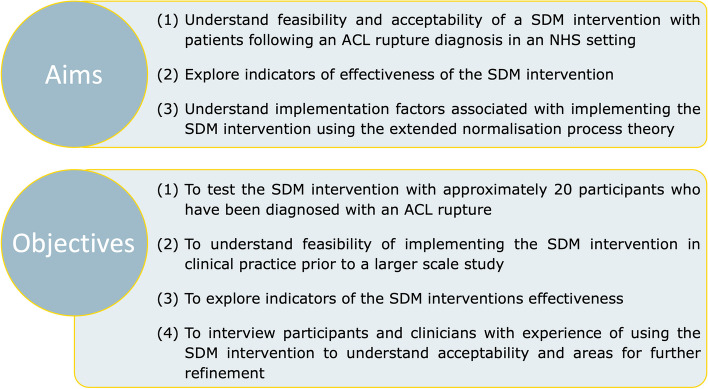
Study aims and objectives

To report the protocol for a mixed-methods feasibility study. The aims and objectives are shown in Fig. 1.

## Methods

### Trial design

**Fig. 2:**
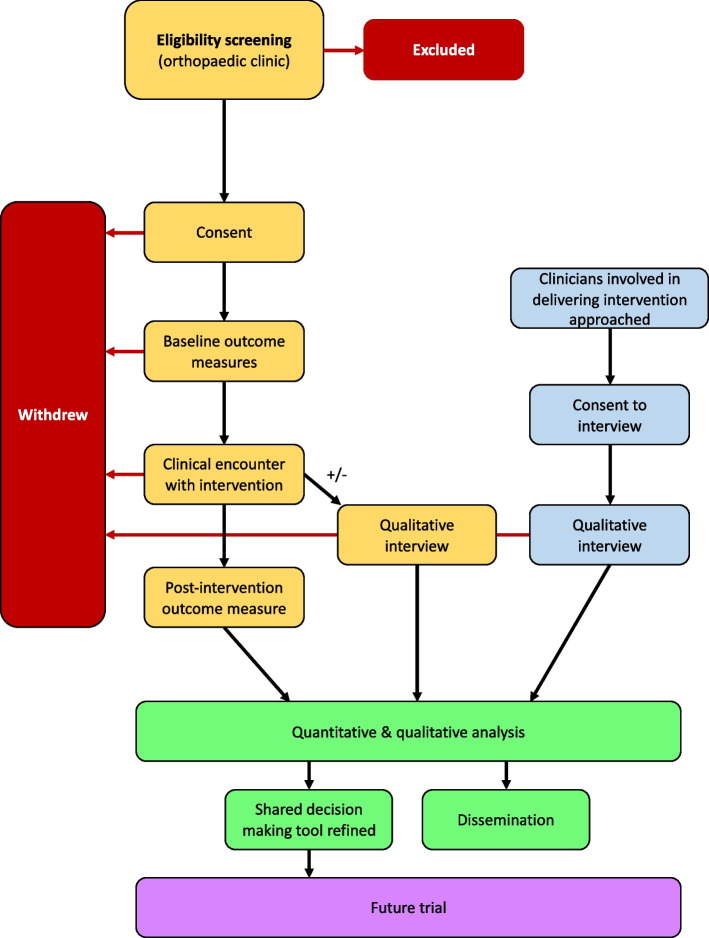
Study flow chart

This is a non-randomised feasibility study with nested qualitative interviews. The study flow chart is shown in Fig. 2. The Standard Protocol Items: Recommendations for Interventional Trials (SPIRIT) statement is available in Additional file 1 [6]. This study will be registered with ISRCTN.

### Study setting

The study will be conducted at one NHS Trust, across three sites, in England providing orthopaedic and outpatient musculoskeletal physiotherapy for adults with an ACL rupture.

### Patient eligibility criteria and identification

Participants will be eligible for inclusion in the feasibility study if they are aged 18 or over and have been diagnosed with an ACL rupture, for the first time in that limb, confirmed by a magnetic resonance imaging (MRI) scan. Exclusion criteria includes those with concomitant injuries requiring surgical intervention that will significantly alter usual treatment, e.g. fracture, bucket handle meniscal tear requiring immediate surgical intervention prior to ACLR, previous surgery to the affected limb or patients who are pregnant (as this is likely to affect decision-making regarding surgical treatment and rehabilitation).

Potential participants will be identified by the orthopaedic team during the patient’s clinical appointment where they are diagnosed with an ACL rupture. They will confirm eligibility and gain consent for the researcher to make contact to discuss the study.

If a patient interested in the study does not read or speak English, relevant study material will be translated into their preferred language and communicated with facilitation of a translator. This will be arranged following normal procedures of the in-house translation service at the University Hospitals of Derby and Burton NHS Foundation Trust.

Clinicians involved in delivering the SDM intervention will be invited to take part in the interviews in addition to the patient participants.

### Recruitment

Patients meeting the eligibility criteria will be invited to participate in the study and provided with the participant information sheet (PIS) and consent documents. There will be the opportunity for potential participants to ask any questions and discuss or clarify information on the PIS, prior to gaining consent. Consent will be gained as per Good Clinical Practice guidelines on paper or via an online database (REDCap electronic data capture tools hosted at the University of Nottingham) [7, 8]. This will include an explanation of the study purpose and what participation in the study involves including its benefits, risks, burdens and rights to withdraw at any time.

Participants willing to be interviewed will provide additional consent to be contacted after their clinical consultation using the SDM tools.

### Sample size

The feasibility trial aims to recruit 20 participants into the study. We will recruit for a maximum of 6 months. This will allow for the project’s objectives to be achieved and is consistent with other UK feasibility trials [9]. The qualitative interviews will be completed with physiotherapists delivering the intervention and the participants receiving it. We aim to recruit 12 participants (patients and clinicians) for the interviews. We estimate that this will be sufficient to reach data saturation and meets the pragmatic objectives of the study [10].

### Intervention

The intervention is a SDM tool. The SDM tool comprises of two parts:

1. Pre-encounter tool
2. Encounter tool

The pre-encounter tool is a patient information leaflet designed for use ahead of the clinical consultation to support increases in patient knowledge. The encounter tool is an option grid designed for use during clinical consultations between patients and clinicians. Training will be provided to physiotherapists to support use of the SDM intervention during clinical consultations.

Other than being provided with the SDM intervention and having a discussion using the option grid during the normal clinical consultation, the participants care will remain the same.

### Outcomes

The primary outcomes of this study are to determine feasibility of a definitive trial. This will involve evaluation of four main outcomes:

1. Recruitment rate
2. Fidelity of intervention delivery
3. Acceptability
4. Follow-up questionnaire completion

Evaluation of these outcomes will be combined with data from qualitative interviews of patients and clinicians involved in the study.

Patient-reported outcomes (PROs) will be collected immediately after the participants clinical consultation where the SDM intervention was used. The PROs to be collected include the following:

1. Acceptability questionnaire
2. Satisfaction with decision (SWD) scale

The SWD scale is the secondary outcome.

### Data collection methods

Once consented, the following baseline data will be collected for each participant:

- Age
- Sex and gender
- Ethnicity
- Postcode
- Highest level of education
- Rapid Estimate of Adult Literacy in Medicine, Revised (REALM-R)
- Months since and mechanism of ACL injury
- Time since diagnosis
- Whether they have been listed for surgery/recommended a treatment by the orthopaedic team
- Preinjury and current activity level
- Employment and current working status

Participants will be encouraged to complete the PROs immediately after their clinical consultation. Participants who fail to do so will receive up to three texts, email, or call reminders (as guided by Patient and Public Involvement and Engagement [PPIE] consultations) 4 weeks after their consultation. Participants will be offered the choice of data collection via paper or online via REDCap.

The participant timeline is shown in Table 1.

**Table 1:** Participant timeline and schedule of events

	Timepoint		
	t_0_	t_1_	Within 4-weeks
Enrolment			
Eligibility screen	X		
Informed consent (*intervention*)	X		
Informed consent (*qualitative study*)	X		
Intervention			
Clinical consultation using SDM tool		X	
Assessments			
Baseline	X		
Follow-up			X
Qualitative interview			X (*approximate*)

### Nested qualitative study

#### Aims

The aim of the nested qualitative study is to support understanding of acceptability of the SDM intervention by patients and clinicians, in addition to understanding contamination and factors associated with implementing the tools in clinical practice. Patient and clinician views on study processes will also be explored to support refinement of the intervention and trial design ahead of a future main trial.

#### Recruitment and sampling

Approximately, 12 participants will be purposively sampled and interviewed. A varied sample will be obtained, in relation to participant characteristics such as age, sex and education level. We aim to recruit 12 participants (patients and clinicians) for the interviews as it is anticipated this will be sufficient to achieve data saturation. Information relating to the interviews will be included in the PIS, and an option to provide consent to be contacted for the interviews will be included in the consent form. After completion of the clinical consultation, if prior consent has been provided, participants will be contacted to confirm interest to participate in the interview, and a suitable date/time/location will be arranged.

#### Data collection

Semi-structured interviews will be completed in person or virtually according to the participants preference. Virtual interviews will be completed via telephone or Microsoft Teams. The topic guides will be informed by ENPT and PPIE input.

Where interviews take place in person, travel and reasonable childcare expenses will be reimbursed. Participants will receive a £20 voucher on completion of the interview.

#### Data analysis

Framework analysis will be used to analyse interview data underpinned by ENPT to explore acceptability, contamination and implementation factors. The CI will keep a reflexive journal to document initial thoughts after each interview and on initial reading of the transcripts. Initial interview data will be mapped to two matrices:

1. Acceptability
2. Implementation and contamination

Sub-headings of each matrix will be decided amongst the study team and with support from PPIE consultations. Each construct of ENPT will be used for matrix 2 (potential/capability/capacity/contribution). Matrices will be refined amongst the study team after mapping of initial interview data as appropriate. Following data mapping onto the two matrices, data will be organised into broad themes in aim to summarise the dataset.

### Data management

Data will be collected using paper and electronic methods, dependent upon participant preference. A patient ID number will be used rather than identifiable information. Data from paper forms will be transcribed into an electronic database in Microsoft Word or Excel stored on OneDrive. Microsoft OneDrive is an ISO 27001 information security management compliant service that allows secure and controlled sharing of data amongst the research team. Data will also be backed up to secure servers at UHDB. Paper hard copies will be stored in the relevant Investigator Site Files. Study documentation will be stored securely (i.e. cupboards, shelves or filing cabinets with restricted access, e.g. within a locked office) to maintain participant confidentiality and study data integrity. Outcome measure data will be collected using software (REDCap) or paper (participant preference). Qualitative data will be organised and managed using NVivo software. Audio recordings and transcriptions will be stored on OneDrive and backed up to secure servers at UHDB. An NHS-approved third-party transcription service will be used that complies with data security regulations. Audio recordings will be uploaded to OneDrive and deleted from the original recording device. Recordings kept on OneDrive will be archived.

### Data analysis and statistical methods

Descriptive statistics will be presented to summarize baseline variables of participants. The categorical variables (e.g. sex, ethnicity) will be reported with frequencies and percentages.

A Consolidated Standards of Reporting Trials (CONSORT) flow diagram will be produced, showing the frequency of patients/participants:

- Assessed for eligibility
- Frequency of each reason for not being eligible
- Found eligible
- Excluded before consent (and the frequency of each reason for exclusion)
- Consented
- Received the intervention (SDM tool) during the clinical consultation
- Lost to follow-up
- Not analysed

The primary outcome data will be analysed as shown in Table 2.

**Table 2:** Primary outcome data

Criteria	Measured
Recruitment rate	Percentage of eligible patients approached to participate
Fidelity	Adherence of delivery will be evaluated by analysis of a case report form (documented by the treating clinician detailing the clinical consultation using the SDM intervention) measured against components of the important details of the SDM tools
Acceptability	Data from the acceptability questionnaire, presented as percentages of agreement to each statement and qualitative data from individual interviews
Follow-up questionnaire completion	Percentage of PROs completed and/or percentage of the forms completed

The feasibility study aims to provide estimates of the recruitment, intervention fidelity, acceptability and follow-up rates to inform a future trial. Feasibility will also be evaluated through qualitative interviews, retention rates and reasons for withdrawal. The estimates will be used in combination with the qualitative data, in discussion with the trial management and independent oversite groups (which includes patient representatives) such as the Trial Steering Committee, to consider success and how the trial may need to be modified to address any shortfalls. Data from the SWD scale will be presented as the number of participants who strongly agree, agree, neither agree nor disagree, disagree and strongly disagree with each statement. In addition, an overall level of satisfaction with decision will be reported (validated scale used: strongly agree [5], agree [4], neither agree nor disagree [3], disagree [4] and strongly disagree [1] [11, 12]), with a higher value indicating a higher satisfaction with decision. SWD data will contribute to understanding of the intervention’s effectiveness, informing intervention refinements and the sample size calculations for the future main trial.

Qualitative interview data will be analysed using a framework approach. Data will be described using themes relevant to the objectives of the interview study.

### Data monitoring and auditing

The site principal investigators (PIs) must ensure that source documents and other documentation for this study are made available to study monitors, the research ethics committee (REC) or regulatory authority inspectors. Authorised representatives of the sponsor (University Hospitals of Derby and Burton NHS Foundation Trust) may visit the participating sites to conduct audits/inspections. The CI will control access to the electronic database. Direct access will be granted to authorised representatives from the sponsor, host institution and the regulatory authorities to permit study-related monitoring, audits and inspections.

### Harms

All adverse events (AEs) and serious adverse events (SAEs) will be recorded and reviewed from the time of informed consent until 4 weeks after the clinical consultation using the SDM tool. All AEs/SAEs occurring during the study will be recorded by the site PI and sent for review by the chief investigator (CI) within 48 h. All related and unexpected SAEs will be reported using the ‘non-CTIMP safety report to REC form’ from the Health Research Authority (HRA) website by the CI. The completed form will be submitted to the sponsor and REC within 15 days of the CI becoming aware of the event. Safety information will be reviewed during trial management group meetings and evaluated by the research team and sponsor at the end of the study.

## Discussion

This study will determine the feasibility, acceptability and indicators of effectiveness for a novel ACL treatment SDM intervention. In the long term, the SDM intervention may improve service and patient outcomes and ensure cost-effectiveness for the NHS; ensuring only those who are most likely to benefit from surgical treatment proceed along this pathway.

The SDM intervention was designed in accordance with the Medical Research Council (MRC) framework for the development of complex interventions, underpinned by ENPT [13]. It was codesigned with patients and relevant stakeholders (including patients, physiotherapists, an occupational therapist, surgeon and outpatient therapy manager) through a nominal group technique consensus method. This involved combining relevant literature and patient, clinician and managerial experience and input.

This study has been designed pragmatically to be delivered in a secondary care NHS setting. Delivery of the SDM tools by a physiotherapist was decided based on capacity across the pathway and the existing skillset of the profession. Implementation factors explored through the qualitative interviews will support future consideration of integrating the tools in practice, to understand who and where the tools could be implemented and by whom. The qualitative and quantitative data will therefore support refinement of the SDM tools and logic model.

In summary, this low-cost intervention seeks to support SDM practices between patients and clinicians making decisions regarding treatment after an ACL rupture. Whilst surgery is common, previous research has demonstrated that it is not successful for all patients, with sub-optimal return to physical activity rates up to and beyond 18 months after surgery [4, 14–16]. In addition, 7.2% are reported to undergo revision surgery within 9 years [17]. Further, understanding patient satisfaction following ACLR or nonsurgical treatment is limited. A 2016 systematic review of 22 studies in US populations sought to examine the quality of patient satisfaction reporting post-ACLR [18]. The review concluded that the level of available evidence was low, and reporting methods were varied across the studies. The authors further noted a decline in reporting of patient-reported satisfaction outcomes in the preceding decade. A 2017 retrospective review of 232 active patients included in a US institutional ACL registry reported 74% to be ‘very satisfied’ 2 years following ACLR, declining to 65.5% at 5 years, with patients more likely to respond ‘very satisfied’ if they had returned to play (*p* < 0.001) [19]. However, limited data exists to understand satisfaction outside this cohort, particularly relevant to the UK context. As return to physical activity outcomes are sub-optimal, patient satisfaction data may support patients and clinicians in understanding outcomes following ACL rupture (managed with surgical and non-surgical intervention) and aid decision-making following rupture.

A systematic review of the SDM literature for people facing health treatment decisions revealed a higher proportion of patients exposed to a patient decision aid reported higher satisfaction with treatment choice [20]. Ensuring patients are on the appropriate pathway has the potential to improve patient outcomes and alleviate service pressures and cost saving for the NHS. This study will support the understanding of feasibility of the intervention to support future trial planning in addition to implementation factors, acceptability and indicators of effectiveness.

## Disclaimer

This paper presents independent research funded by the Health Education England (HEE)/NIHR for this research project. The views expressed in this publication are those of the author(s) and not necessarily those of the NIHR, NHS or the UK Department of Health and Social Care.

### Supplementary Information

Supplementary Material 1. SPIRIT 2013 Checklist: Recommended items to address in a clinical trial protocol and related documents

## Data Availability

Data sharing is not applicable to this article as no datasets were generated or analysed during the current study.

## Abbreviations

AE: Adverse events
ACL: Anterior cruciate ligament
ACLR: Anterior cruciate ligament reconstruction
CI: Chief investigator
CONSORT: Consolidated Standards of Reporting Trials
ENPT: Extended normalisation process theory
IKDC: International Knee Documentation Committee
KOOS: Knee Injury and Osteoarthritis Outcome Score
MRC: Medical Research Council
MRI: Magnetic resonance imaging
QoL: Quality of life
PIs: Principal investigators
PIS: Participant Information Sheet
PPIE: Patient and Public Involvement and Engagement
PROs: Patient-reported outcomes
REALM-R: Rapid Estimate of Adult Literacy in Medicine, Revised
REC: Research Ethics Committee
RCT: Randomised controlled trial
SAE: Serious adverse events
SDM: Shared decision-making
SPIRIT: Standard Protocol Items: Recommendations for Interventional Trials
SWD: Satisfaction with Decision Scale
